# The potential for an outbreak of glanders in Nepal

**DOI:** 10.1186/s41182-019-0185-2

**Published:** 2019-12-11

**Authors:** Niran Adhikari, Krishna Prasad Acharya, Richard Trevor Wilson

**Affiliations:** 1 Animal Health Training & Consultancy Services, Pokhara, Nepal; 2 Animal Quarantine Office (AQO), Budhanilkantha, Kathmandu Nepal; 3 Bartridge House, Umberleigh, UK

**Keywords:** Equine diseases, Farcy, *Burkholderia mallei*, Mallein test, Re-emerging diseases

## Abstract

Confirmation of glanders has not been possible in suspected cases submitted by field veterinarians, mainly due to the lack of diagnostic tools in Nepal. In view, however, of the re-emergence of glanders in India and the unrestricted migration of equines from there in to Nepal, an outbreak of Glanders in the short term is a distinct possibility. Such an event would affect the rural, marginalized community, and brick kiln industries. Therefore, due attention on the national epidemiological study and strengthened animal quarantine system with holding yards and laboratory backups are highly requested. Besides, the government’s timely action on disease prevalence, monitoring, and disease reporting is utmost important besides widespread public awareness to prevent the entry and control the disease.

Glanders or farcy is a rare, contagious, infectious zoonotic disease that is caused by the bacterium *Burkholderia mallei* [1]. It mainly affects horses, mules, and donkeys. The late fifteenth-century French “glandres” and various other historical names underline the former importance of this disease. Symptoms include nasal discharge, pneumonia, and ulcerating nodular lesions on the skin. The disease is transmitted via contaminated feed, water, and meat. It is mainly reported from areas where equids are housed under unhygienic, overcrowded, and stressful environments with practices of sharing grooming equipment, water holes, and grazing areas [2]. Glanders used to be global problem but was later eradicated from Europe, North America, and Australia by collaboration among governments and other agencies. Glanders has regained global attention as a re-immerging disease with confirmed cases from Bahrain, Germany, [3, 4] and various parts of India [5].

The first confirmed case of glanders in equines in India was in 1913 and was later reported from several areas before being brought under control [6]. Glanders was re-introduced to India in 1962 with the import of unscreened equines during the Indo-Chinese war. Numerous cases have since been reported [2]. Glanders reappeared in Maharashtra State in July 2006 and has subsequently been reported in several states. In the period 2006–2017, the disease’s outbreak increased by more than 100%. [5] (Table 1). Uttar Pradesh, from which most equines and most of immigrant laborers arrive in Nepal [7], recorded the highest number of Glanders outbreaks (Fig. 1) with significant records during March–July (Table 1) [5] which coincides with the months of the greatest number of horse imports to Nepal [8]. No confirmed cases of glanders have so far been identified but the threat of the disease being introduced is very real in view of the weak surveillance and animal quarantine activities.

**Table 1:** Recorded number of Glanders outbreak in India, 2006–2017

Month	Year									
	2006	2007	2008	2009	2010	2011	2012	2015	2016	2017
January						3 (U)			1 (G)	1 (J) +1 (U)
February									1 (J)	1 (J) + 3 (U)
March					1(C)		1 (U)	1 (H)+ 1 (J)	4 (G) + 1 (J)	1 (J) +1 (G)+ 4 (U)
April		+() (W)	+() (W)	+() (W)						8 (U)
May									1 (J)	1 (J) +1 (G)+ 2 (M) +9(U)
June					1 (H)				1 (J)	1 (J) + 13 (U) + 2 (R)
July	2 (M)								1 (J)	6(U)+1(M)
August										9(U)
September					+() (W)	+() (W)				21(U)+1(R)
October										3(M)+1(J)
November										10(U)+1(J)
December										5(U)

+ (): Disease limited to one or more zones, *G* Gujarat, *C* Chhattisgarh, *H* Himachal, *J* Jammu & kashmir, *M* Maharastrha, *R* Rajasthan, *U* Uttarpradesh, *W* Whole Country

**Fig 1:**
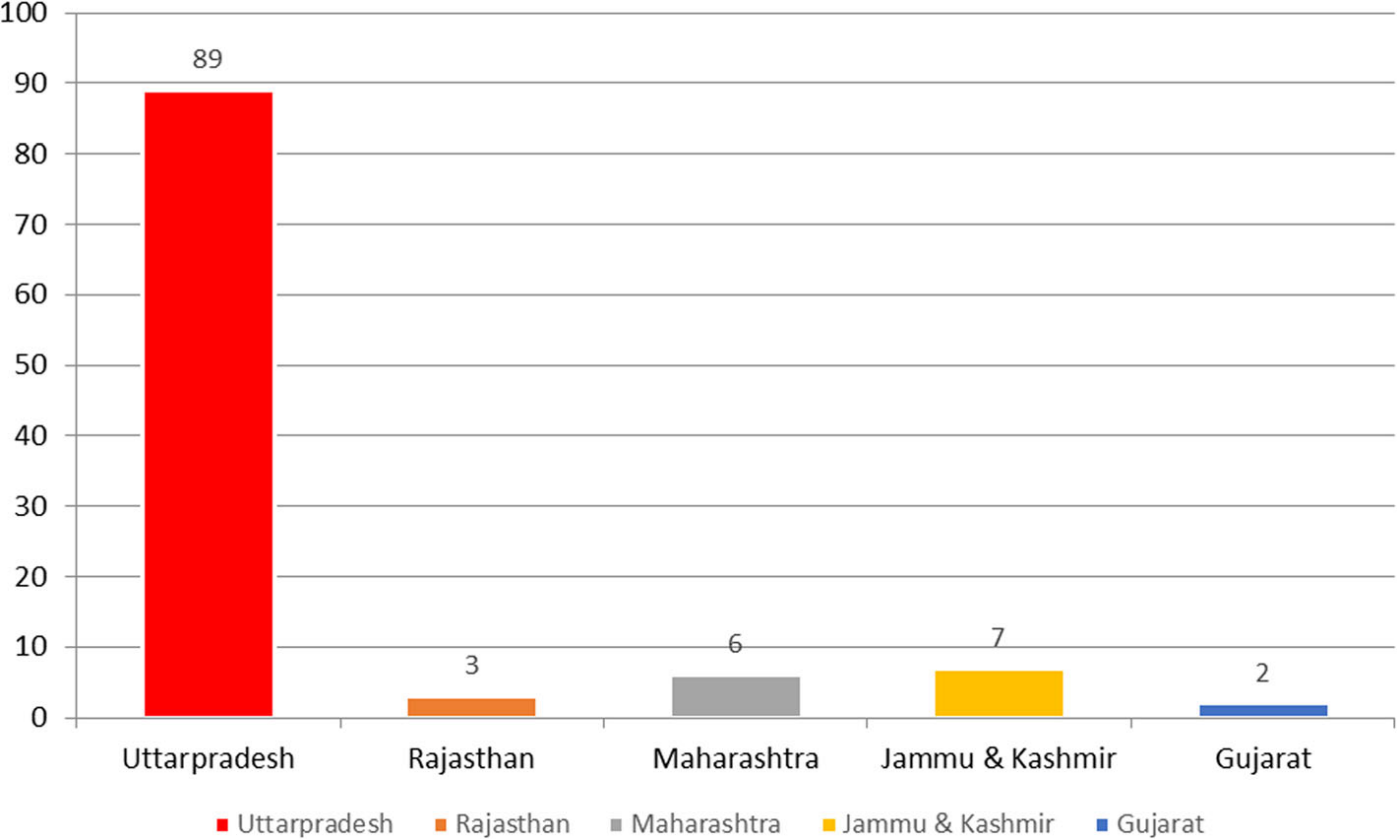
Recorded number of Glanders outbreak in equines in Indian regions, 2017

Glanders was listed as one of the contagious diseases of equines (B209) in the Animal Health and Livestock Services Act, 2055 [9] after which quarantine offices were established. In 2019, Nepal has eight quarantine offices and 29 animal quarantine check-posts. The annual report of the Central Animal Quarantine Office indicates that Nepal imported 2690 equines from India in 2073/74 the year AD 2017. Most equines imported from India passed through the Nepalgunj quarantine office, the closest to India’s Uttar Pradesh region. Quarantine officers can prohibit entry of animal from such disease outbreak regions but poor coordination with Indian quarantine check posts, lack of animal holding yards, and illegal migration through the open border create problems for the Nepalese quarantine service.

Nepal’s Central Veterinary Laboratory (CVL) is the national veterinary reference laboratory. Samples from suspicious imported animals are dispatched to the CVL for analysis [10]. In 2071/72 (AD 2014), CVL conducted epidemiological investigations and surveillance of animal diseases but does not have the facility to test for glanders [11]. The guidelines of the World Organization for Animal Help (Office International des Epizooties, OIE) state that a standard cultural and serological test is required for glanders [12]. The Complement Fixation Test (CFT) has a sensitivity of 91.4%, a specificity of 100% and an accuracy of 96.7%. It can detect glanders in both clinically unapparent carriers and chronically infected equines but is incompetent with anti-complementary equine serum [13]. Unfortunately, its high cost means it is less affordable as a screen test for low-income countries such as Nepal.

Isolation and identification of *B. mallei* isolation requires a bio-safety level 3 (BSL3) laboratory [14, 15]. The National Public Health Laboratory (NPHL) is the only BSL3 laboratory in Nepal but largely confines itself to human diseases [16]. Possible tests for glanders are the culture test but this is negative before death in the septicemic form of the disease. The Mallein test is simple, has a sensitivity of 75.57%, a specificity of100% and an accuracy of 90.6 % [13], but is less efficient in clinically advanced cases poor competence in technicians may cause injury and blindness to the animal. The Rose Bengal test (RBT) is another similar affordable screening test with a sensitivity of 90%, a specificity of 100% and an accuracy of: 96.1% [13], but unlike the Mallein test, it is more potent in cases of non-response or advanced glanders [17]. The best approach to identification of glanders in Nepal would appear to be a Mallein or RBT test as a screen test at quarantine check posts and CFT as confirmative diagnosis at the CVL.

Equines have contributed to human welfare and people’s livelihoods in Nepal since time immemorial. Horses especially, but also donkeys and mules, are a major means of transport in rural areas. They are also used by the Nepalese Army and Police Force and are a major component of the brick-making industry in southern Nepal [18] where more than 2200 animals of the 56,834 Nepali equine populations work [19]. Brick-making is one of the largest employers of human labor in the country. An outbreak of glanders would thus have an enormous negative impact not only on the horses but also on the poor people who work with them and have no possibility of other forms of employment [7].

## Conclusion

Consequent on the re-emergence of Glanders in India and periodic unrestricted migration of equines from there and especially from Uttar Pradesh neighboring on Nepal an outbreak of Glanders in Nepal is a distinct possibility. Such an outbreak would have an adverse impact not only on Nepal’s equine population but also on the welfare and livelihoods of a large number of poor families and especially those working in the brick-making industry. More attention by government to the disease is warranted. Quarantine offices should keep full records of equines imported from outbreak regions in and possibly prohibit such entry. Where equines are allowed entry effective short-term measures to prevent or control glanders would be screening of imported animals with the Mallein or RBT test at quarantine check posts followed by a CFT test at the CVL. The long-term goal should be to improve coordination between quarantine offices on both sides of the border zone, enforce stringent quarantine measures, test for glanders in resident equines in Nepal, euthanasia positive glanders cases, and create greater awareness by the general public of the disease and its potential impact through specific campaign and in the media.

## Data Availability

The datasets generated and/or analyzed during the current study are available in the OIE World Animal Health Information System repository, *https://www.oie.int/wahis_2/public/wahid.php/Diseaseinformation/statusdetail. Accessed 23 Sep 2019.* Not applicable (NA).

## Abbreviations

BSL3: Bio-safety level 3
CFT: Complement fixation test
CVL: Central Veterinary Laboratory
NPHL: National Public Health Laboratory
OIE: Office International des Epizooties
RBT: Rose Bengal test
